# U. S. Pharmacopœia

**Published:** 1849-07

**Authors:** Geo. B. Wood

**Affiliations:** Vice-President of the Convention of 1849; Philad.


### ARTICLE V.

*National Convention for revising the Pharmacopœia of the United States.*

The Convention for revising the Pharmacopœia, which met in Washington, in January 1840, adopted the following resolutions.

"1st. The President of this Convention shall, on the first day of May 1849, issue a notice requesting the several *incorporated State Medical Societies, the incorporated Medical Colleges, the incorporated Colleges of Physicians and Surgeons and the incorporated Colleges of Pharmacy*, throughout the U. S., to elect a number of delegates not exceeding three, to attend a general Convention to be held at Washington, on the first day in May, 1850.

"2. The incorporated bodies, thus addressed, shall also be requested by the President to submit the Pharmacopœia to a careful revision, and transmit the result of their labours, through their delegates, or any other channel, to the next Convention.

"3. The several medical and pharmaceutical bodies shall be further requested to transmit to the President of the Convention, the names and residences of their respective delegates as soon as they shall have been appointed, a list of whom shall be published, under his authority, for the information of the medical public, in the newspapers and Medical Journals, in the month of March, 1850.

"4. In the event of the death, resignation, or inability to act of the President of the Convention, these duties shall devolve on the Vice-President, and, should the Vice-President also be prevented from serving, upon the Secretary or Assistant Secretary, the latter acting in the event of the inability of the former."

In compliance with the foregoing resolutions, the undersigned, having been informed by the President of the late

Convention, Dr. Lewis Condict, that he would be unable, from indisposition, to perform the duties assigned to him, gives notice to the several medical and pharmaceutical bodies enumerated in the first resolution, that a Convention for revising the National Pharmacopœia, will meet in the City of Washington, on the first Monday in May, 1850. The undersigned also requests of the several bodies referred to, that they will fulfil the wishes of the Convention, set forth in the second resolution; and, further, that they will transmit to his address, on or before the 1st of March next, the names and residences of the delegates whom they may appoint, in order that a list of them may be published, as directed in the third resolution.

GEO. B. WOOD, M. D.,
Vice-President of the Convention of 1849.

*Philad.*, *May 1st*, 1849.

